# Characteristic Optical Coherence Tomography and Magnetic Resonance Imaging Findings in a Case of Seronegative Neuroretinitis

**DOI:** 10.7759/cureus.95233

**Published:** 2025-10-23

**Authors:** Camden E Treat, Noah Vermette, Shamseldeen Mahmoud

**Affiliations:** 1 Radiology, Saint Louis University School of Medicine, St. Louis, USA

**Keywords:** magnetic resonance imaging, neuroradiology, neuroretinitis, ophthalmology, optical coherence tomography, vision loss

## Abstract

Neuroretinitis is a rare ophthalmologic disorder that presents with painless loss of vision. In this report, we describe the magnetic resonance imaging (MRI) findings in a case of seronegative, idiopathic neuroretinitis, which occurred in the setting of a recent tick bite. A 42-year-old man with no past medical history of eye complaints presented to Saint Louis University Emergency Department with painless loss of visual acuity in his left eye worsening over the span of one week. The patient denied additional ocular symptoms and any symptoms of recent infection such as headache, fever, cough, sore throat, chest pain, joint pain, nausea, and vomiting. Relevant recent history was limited to a report of a tick bite while traveling in Illinois that was followed by a self-resolving “red ring” rash. However, infectious workup, including tests for Lyme disease antibody, was negative. An MRI of the brain and orbits with and without contrast was performed, demonstrating increased thickening and enhancement in the left optic disc. Ocular coherence tomography (OCT) demonstrated optic nerve edema with intraretinal fluid with scattered exudates extending to fovea, along with distortion of contour of subretinal fluid. These imaging findings, in conjunction with patient history and fundoscopic examination, supported a diagnosis of neuroretinitis with no identified etiology.

## Introduction

Neuroretinitis (NR) is an uncommon ocular condition caused by inflammation of both the optic nerve and retina. Clinically, this condition often presents as an acute, unilateral, painless loss of vision, often accompanied by dyschromatopsia and scotoma. Diagnosis of NR may be made through a fundoscopic examination revealing the presence of macular exudates radiating from the fovea to form the characteristic “macular star” [1]. This finding elucidates the underlying pathophysiology of NR, which involves inflammation-induced permeability of the optic disc, allowing fluids and lipids to accumulate in the plexiform layer of the retina. It is important to distinguish NR, which involves inflammation in both the optic nerve and retina, from optic neuritis, a condition where only the optic nerve is affected and the fundoscopic exam remains normal.

The etiology of NR may be idiopathic or infectious, with cat-scratch disease NR caused by *Bartonella henselae* accounting for up to two-thirds of cases [2]. Other infectious causes include syphilis, Lyme disease, toxoplasmosis, and viral infections [3]. In this case report, we present the magnetic resonance imaging (MRI) findings of idiopathic NR. This case demonstrates a unique clinical scenario in which the patient exhibited classic symptoms of NR with a negative infectious workup and nonspecific fundoscopic findings, necessitating radiological evaluation with MRI. 

## Case presentation

A 42-year-old previously healthy man with no past medical history presented to the emergency department with concern for vision changes in the left eye over the course of one week. These vision changes were described as a painless and slowly progressive loss of visual acuity with "blurred" vision. Earlier that day, the patient had presented to an optometry clinic, where a fundoscopic examination demonstrated optic nerve inflammation with hemorrhage, prompting an instruction to visit the emergency department. 

At presentation, the patient denied constitutional symptoms, including headache, fever, cough, sore throat, chest pain, joint pain, nausea, and vomiting. He also denied ocular-specific symptoms, including floaters in vision, pain with eye movement, eye discharge, or recent eye trauma. The only relevant history included a recent tick bite while on a trip to Illinois, with a subsequent “red ring” rash that self-resolved. The described visual changes began after resolution of the rash.

**Figure 1:**
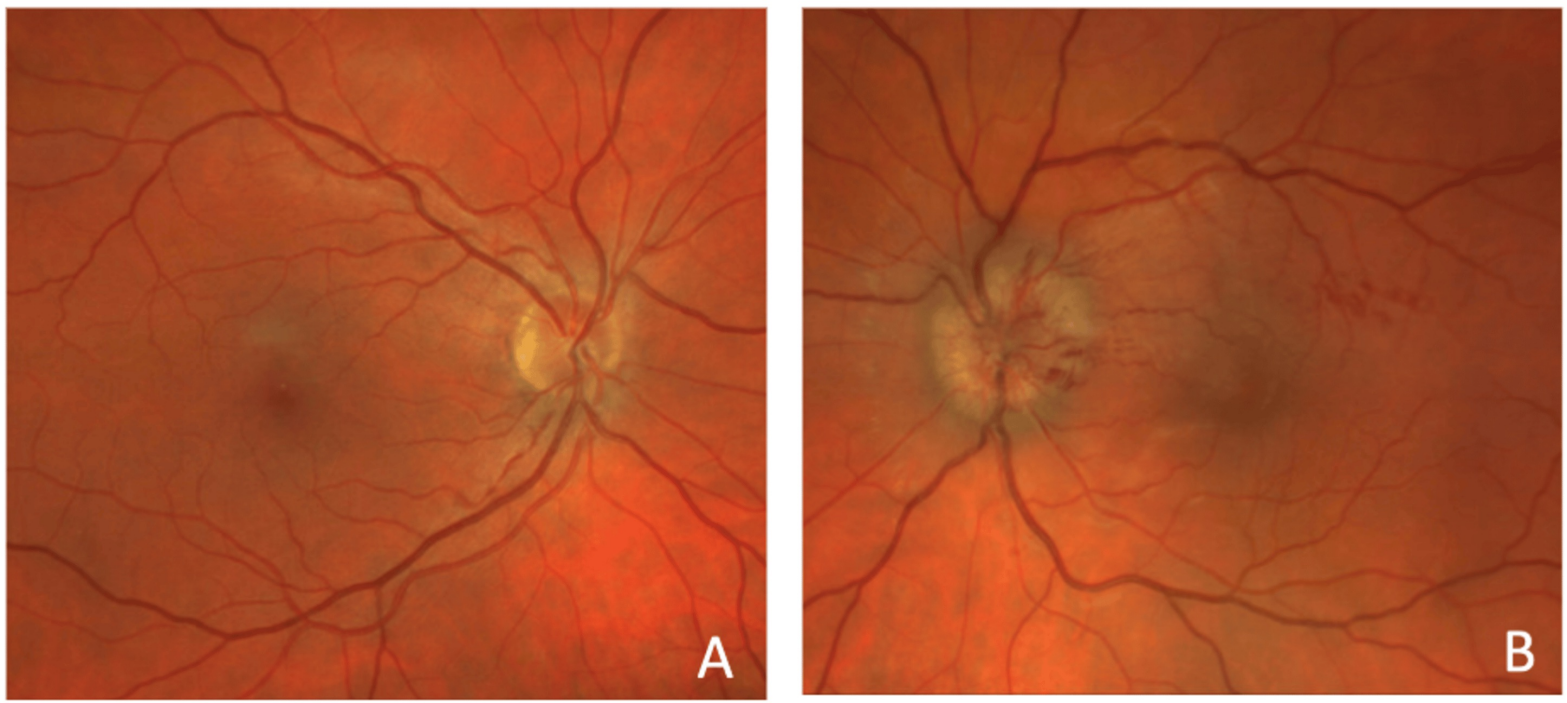
Fundoscopic examination demonstrating a normal right eye (A), and optic nerve edema, peripapillary retinal nerve fiber layer hemorrhages with exudates, and macular edema of the left eye (B).

On initial ophthalmologic examination, visual acuity by Snellen linear chart was 20/20 in the right eye and 20/70 in the left eye. There was a notable afferent pupillary defect on the left, indicating an issue with the optic nerve. Intraocular pressures were normal. Fundoscopic examination (Figure 1) revealed a normal retinal appearance of the right eye (Figure 1A) and optic nerve edema, hemorrhages, and macular edema of the left eye (Figure 1B) concerning for papilledema, ischemic optic neuropathy and other inflammatory optic disc conditions. Initial diagnostic laboratory evaluation results and reference values were recorded (Table 1). In summary, routine bloodwork, including a complete blood count (CBC), comprehensive metabolic panel (CMP), C-reactive protein (CRP), erythrocyte sedimentation rate (ESR) and infectious serology, were all negative. Additional autoimmune workup was not obtained during this patient's emergency department visit.

**Table 1:** Summary of initial laboratory results with corresponding reference ranges. QuantiFERON-TB Gold Plus: blood-based interferon-gamma release assay (IGRA) to detect *Mycobacterium tuberculosis, *Ab: antibody, IgG: immunoglobulin G, L: liter, mL: milliliter, dL: deciliter, mg: milligram, mmol: millimolar, mm: milligram, h: hour.

Lab	Normal Range	Patient’s Value
Sodium	136-145 mmol/L	137 mmol/L
Potassium	3.5-4.5 mmol/L	4.0 mmol/L
Chloride	98-107 mmol/L	105 mmol/L
Carbon dioxide	22-29 mmol/L	24 mmol/L
Creatinine	0.71-1.16 mg/dL	1.02 mg/dL
Blood urea nitrogen	7-26 mg/dL	13 mg/dL
White blood cell count	4,000-10,700x10^9^/L	7,800x10^9^/L
Red blood cell count	4,300-5,800x10^9^/L	5,050x10^9^/L
Hemoglobin	13.3 - 17.5 g/dL	16.0 g/dL
Platelet count	150-420x10^9^/L	285 x 10^9^/L
C-reactive protein	<0.5 mg/dL	<0.5 mg/dL
Erythrocyte sedimentation rate (ESR)	0-15 mm/h	2 mm/h
QuantiFERON-TB Gold Plus	Negative	Negative
Lyme Disease Ab Total	Negative	Negative
*Treponema pallidum* Ab	Negative	Negative
*Bartonella henselae* IgG Ab	Negative	Negative
*Bartonella henselae* IgM Ab	Negative	Negative

**Figure 2:**
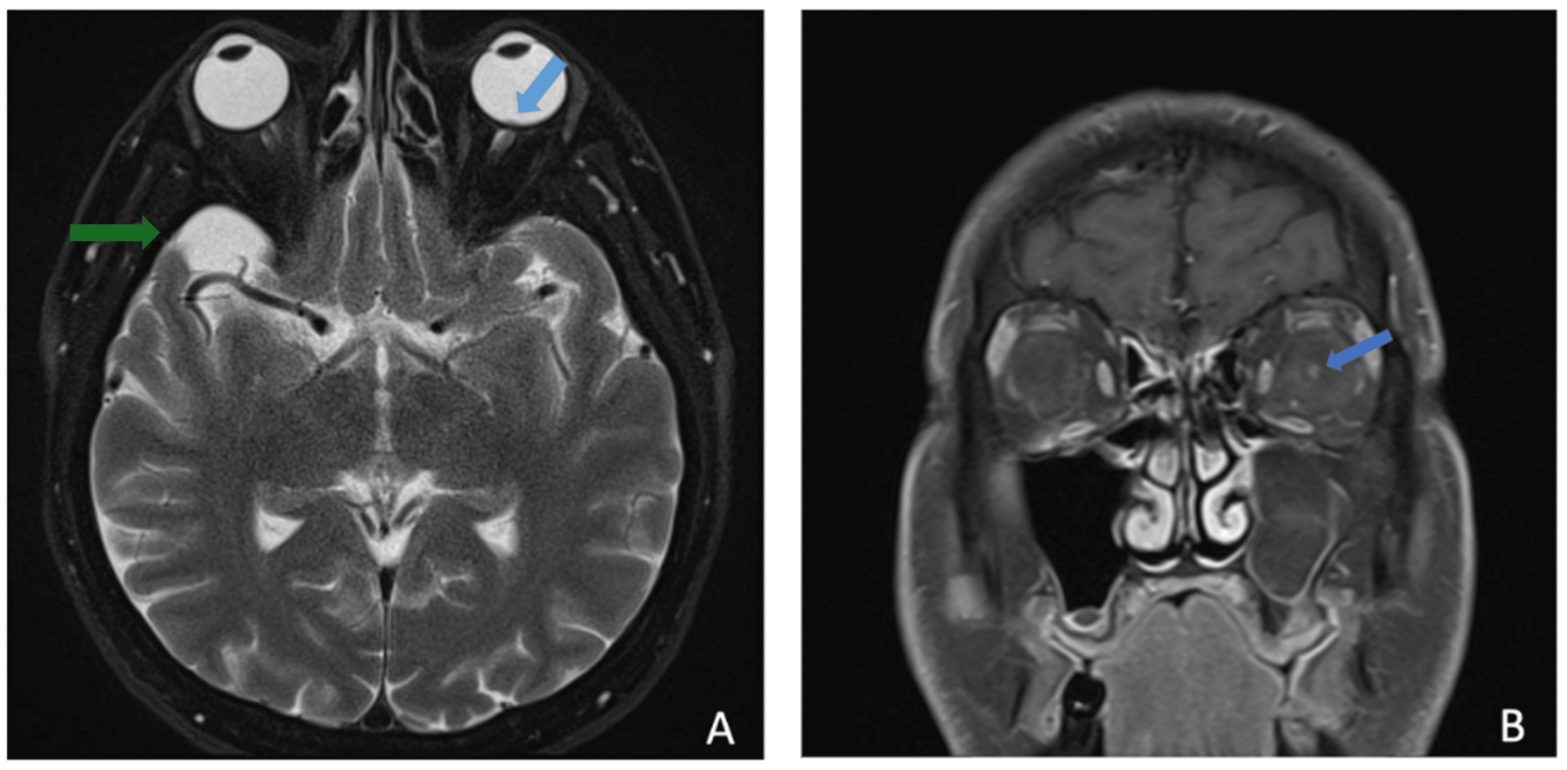
(A) Postcontrast axial T2 fat-saturated blade thin sequence demonstrating bulging of the left optic disc (blue arrow). An incidental arachnoid cyst is visualized in the right temporal lobe (green arrow). (B) Postcontrast coronal T1 fat-saturated thin sequence demonstrating left optic disc enhancement (blue arrow).

**Figure 3:**
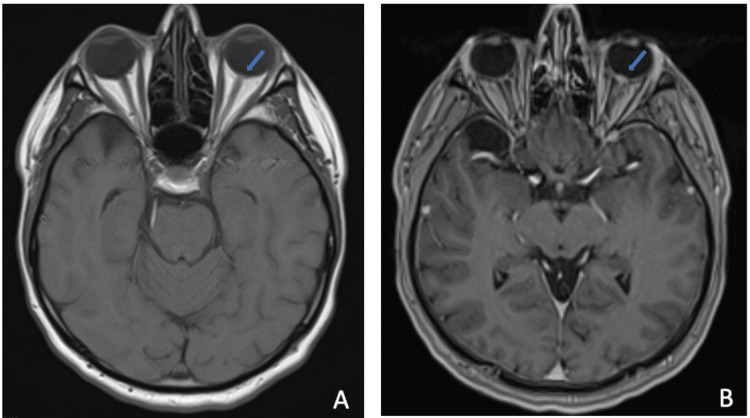
(A) Precontrast axial T1 thin sequence demonstrating lack of enhancement of left optic disc (blue arrow). (B) Postcontrast axial T1 fat-saturated thin sequence demonstrating left optic disc enhancement, suggesting inflammation (blue arrow).

**Figure 4:**
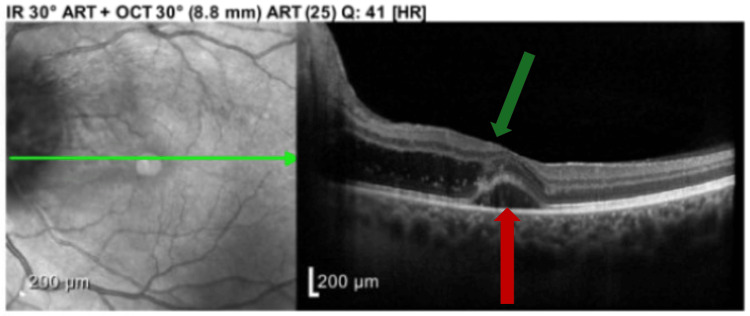
Ocular coherence tomography (OCT) demonstrating optic nerve edema (green arrow) with intraretinal fluid (red arrow) with scattered exudates extending to fovea, along with distortion of contour of subretinal fluid.

Given the patient’s nonspecific initial examination and negative laboratory results, an MRI of the brain and orbits with and without contrast was performed for further evaluation. The relevant findings are detailed below (Figures 2, 3) and briefly revealed left optic disc enhancement, suggestive of an underlying inflammatory process. An incidental left-sided maxillary sinus obstruction was visualized on postcontrast MRI, suspected to represent a mucocele (Figure 4). Following the MRI results, the ophthalmology team elected to perform ocular coherence tomography (OCT) (Figure 5), revealing optic nerve edema with the presence of subretinal fluid.

The patient was prescribed a six-week course of doxycycline, given the concern for potential underlying Lyme disease with negative serology and was instructed to follow-up with the ophthalmology clinic as an outpatient for further management. Over the subsequent six weeks of documented follow-up at the time of this report, the patient visited the ophthalmology clinic at one week and three weeks after initial presentation. Repeat visual acuity testing demonstrated improvement to 20/40 in the left eye, which has not yet returned to baseline. Further imaging has not been obtained at the time of this report.

## Discussion

The prognosis of NR is often dependent upon the underlying etiology and availability of targeted treatment. Typically, optic disc edema is a self-limited process with a high recovery rate. One study reports that over 68% of patients with idiopathic or cat-scratch-disease-related NR achieve a final visual acuity of at least 20/40 [4]. Recurrence of NR related to chronic etiologies such as sarcoidosis, Behçet disease, or viral reactivations of varicella zoster virus (VZV) and herpes simplex virus (HSV) has a worse prognosis due to cumulative damage to the optic disc [5]. In cases of recurrent NR, final visual acuity better than 20/40 is achieved in only 36% of patients [1]. This discrepancy in prognosis demonstrates the importance of identifying the cause of NR and promptly initiating case-specific treatment. Due to the broad range of causes, treatment for NR may include no treatment, broad-spectrum antibiotics, organism-specific antibiotics, or high-dose oral corticosteroids [5]. Involvement of medical specialists from infectious disease, ophthalmology, rheumatology, and neurology may also be necessary depending on individual patient history. 

Our patient initially came to the emergency department with complaints of unilateral, painless, slowly progressive loss of visual acuity, and physical examination demonstrated a unilateral afferent pupillary defect. Approximately 67.5% of NR cases present with an afferent pupillary defect as a manifestation of the underlying inflammation of the optic nerve [1]. While our case fits the typical case presentation for NR, patients may also present with diminished color vision, photophobia, and headaches. Infectious and noninfectious NR typically have similar symptoms, so careful consideration of patient history, physical examination, including a thorough skin examination for characteristic rashes, and an infectious workup.

In this case, an infectious etiology was not identified; however, the patient’s history of a tick bite followed by a “red ring” rash raised sufficient clinical suspicion for seronegative Lyme disease to warrant treatment with an empirical course of doxycycline. Early cases of Lyme disease are known to present without positive antibody testing, and antibody testing is not necessary to make the diagnosis in the presence of the characteristic erythema migrans rash [6]. Although it is possible to obtain further testing with high-sensitivity enzyme-linked immunosorbent assay (ELISA) and Western blot testing, this was not performed in the emergency department setting [6].

On fundoscopic examination, macular exudates were present without the classic “macular star,” although this finding is nonspecific and may be associated with hypertensive retinopathy, diabetic papillopathy, papilledema, anterior ischemic optic neuropathy, and other conditions that cause macular exudates. Generally, these systemic causes present with bilateral loss of visual acuity and necessitate evaluation of patient blood pressure, intracranial pressure, and medical history. Features such as bilateral loss of visual acuity, severe eye pain, or retinal vascular occlusion may reduce suspicion for NR and guide the clinician toward investigating other ophthalmologic conditions [3]. 

While imaging is not strictly required for the diagnosis of NR, due to the wide range of presenting symptoms, etiologies, and clinical mimics, imaging can serve as a definitive diagnostic tool. Optical coherence tomography (OCT) is often used because of its ability to capture the presence of subretinal fluid and intraretinal edema prior to the development of macular exudates visible on fundoscopic examination. In cases where a macular star is not identified on fundoscopic examination, OCT may be used for early identification of the exudative process taking place within the retina [7].

In this case, OCT identified optic nerve edema with intraretinal fluid and scattered exudates extending to fovea, which provided additional support for the initial MRI findings suggesting NR. Additionally, OCT can reveal cell collections in the vitreous humor anterior to the optic disc, a finding indicative of an infectious or inflammatory process taking place within the optic disc as opposed to noninflammatory etiologies such as papilledema and ischemic optic neuropathy [8]. Finally, OCT is an excellent method for quantifying the amount of subretinal and intraretinal fluid, and monitoring the resolution of this finding with serial OCT may serve as a prognostic tool for likelihood of visual recovery.

MRI may be preferred over OCT in cases where intracranial sources of optic nerve inflammation or compression must be ruled out. For our case, postcontrast T2FS MRI of the brain and orbits revealed thickening and enhancement of the left optic disc, and postcontrast T1FS MRI showed enhancement near the left retinal optic disc. While many cases in the literature have described NR with a normal MRI, both optic disc enhancement and optic nerve enhancement are expected findings [9].

Our patient’s MRI revealed the presence of a maxillary sinus mucocele ipsilateral to the affected eye. Although no clear connection between mucoceles and NR has been reported in the current literature, sinus mucoceles may be identified as a source of optic nerve compression and subsequent optic neuritis [10]. However, with consideration for the anatomical relation of the maxillary sinus mucocele to the optic nerve, it remains an unlikely source of compression in the patient described here. Although some diagnostic uncertainty remains due to the absence of a clear etiology of this NR case, MRI served as a useful tool in supporting the diagnosis as clear signs of left optic disc and optic nerve inflammation were captured.

## Conclusions

In this case of idiopathic NR, the characteristic imaging findings of NR, including thickening and enhancement of the optic disc and optic nerve were seen on MRI. These findings correlated with both the patient’s symptoms of painless vision loss and presence of combined optic nerve and macular edema seen on fundoscopic examination. Awareness of these characteristic imaging patterns is important for radiologists, as it can lead to timely diagnosis and facilitate early initiation of treatment in patients presenting with acute, painless vision loss.
